# Single-dose trastuzumab monotherapy achieved pathological complete response (pCR) in a patient with HER2-positive breast cancer: a case report

**DOI:** 10.1186/s40792-023-01661-4

**Published:** 2023-06-21

**Authors:** Ayaka Isogai, Haruru Kotani, Masataka Sawaki, Masaya Hattori, Akiyo Yoshimura, Ayumi Kataoka, Kazuki Nozawa, Yuri Ozaki, Yuka Endo, Akira Nakakami, Rie Komaki, Hiroji Iwata

**Affiliations:** Department of Breast Oncology, Aichi Cancer Center Hospital, 1-1 Kanokoden, Chikusa-Ku, Nagoya, 464-8681 Japan

**Keywords:** Breast cancer, HER2-positive, Trastuzumab, Monotherapy, Single dose

## Abstract

**Background:**

With advances in breast cancer treatment, the importance of de-escalation therapy to reduce harm during the treatment of elderly patients has attracted attention in recent years. Certain patient populations are expected to have a superior response to anti-HER2 drugs, particularly those with human epidermal growth factor receptor type 2 (HER2)-positive breast cancer. In this report, we describe our experience of dramatic anti-HER2 drug response in a patient who achieved pathological complete response (pCR) with a single dose of trastuzumab.

**Case presentation:**

An 88-year-old woman presented with a 2-cm palpable mass in the left breast. Vacuum-assisted breast biopsy, ultrasonography, and positron emission tomography–computed tomography revealed estrogen receptor-negative and HER2-positive, T1N0M0, stage I breast cancer. Mastectomy was scheduled within 2 months of the initial visit; however, the patient was anxious about the length of the waiting period and requested medication in the interim. Therefore, prior to surgery, one cycle of trastuzumab monotherapy was administered at the discretion of the attending physician. Postoperative pathology showed no remnant of invasive carcinoma and pCR with only a 0.2-mm ductal carcinoma in situ remnant. The patient refused further medication after surgery because of severe diarrhea after trastuzumab administration. Postoperative treatment was limited to follow-up, and no recurrence was observed at 1 year and 6 months postoperatively.

**Conclusion:**

This case suggests that trastuzumab monotherapy may be effective in certain patients with HER2-positive breast cancer. In the future, identifying patients who are more likely to respond to trastuzumab, as in this case, will allow for more options regarding de-escalation therapy without chemotherapy, particularly in elderly patients who are concerned about the side effects of chemotherapy.

## Background

Breast cancer is the most common cancer in women and one of the most common causes of death in this sex [1]. Overexpression of the human epidermal growth factor receptor 2 (HER2) protein, amplification of the HER2 gene, or both, occurs in approximately 15–25% of breast cancers [2, 3]. A standard systemic therapy for HER2-positive primary breast cancer is a combination of anti-HER2 therapy (e.g., trastuzumab or pertuzumab) and chemotherapy, which is associated with a higher response rate, as anti-HER2 therapy has significantly improved disease-free survival [4–6].

In elderly patients, the aim of treatment is to achieve a beneficial effect with minimal harm. However, chemotherapy may be harmful to elderly patients [7]. Recently, de-escalation therapy without chemotherapy has been considered to reduce the adverse effects of treatment in the elderly population. In the RESPECT study comparing trastuzumab monotherapy with trastuzumab + chemotherapy after surgery, disease-free survival did not meet the hazard ratio, but the loss of observed survival for monotherapy was < 1 month at 3 years. Moreover, monotherapy resulted in fewer adverse events and a more favorable health-related quality of life than trastuzumab + chemotherapy [8]. In the NeoSphere trial, the efficacy of treatment was compared between patients treated with anti-HER2 drugs alone (trastuzumab + pertuzumab) and those treated with anti-HER2 drugs combined with chemotherapy (combination group). The rate of pathological complete response (pCR) in the breast was lower in the trastuzumab + pertuzumab group than that in the combination group. Although the rate of pCR was only 16.8% (95% CI: 10.3–25.3), it can be expected that a certain population will have a superior response to anti-HER2 drugs. Here, we report the case of a patient with a dramatic response to an anti-HER2 drug.

## Case presentation

The patient was an 88-year-old woman with no relevant medical history. A mass was observed in the left breast. She underwent a vacuum-assisted breast biopsy and was diagnosed with breast cancer.

**Fig. 1:**
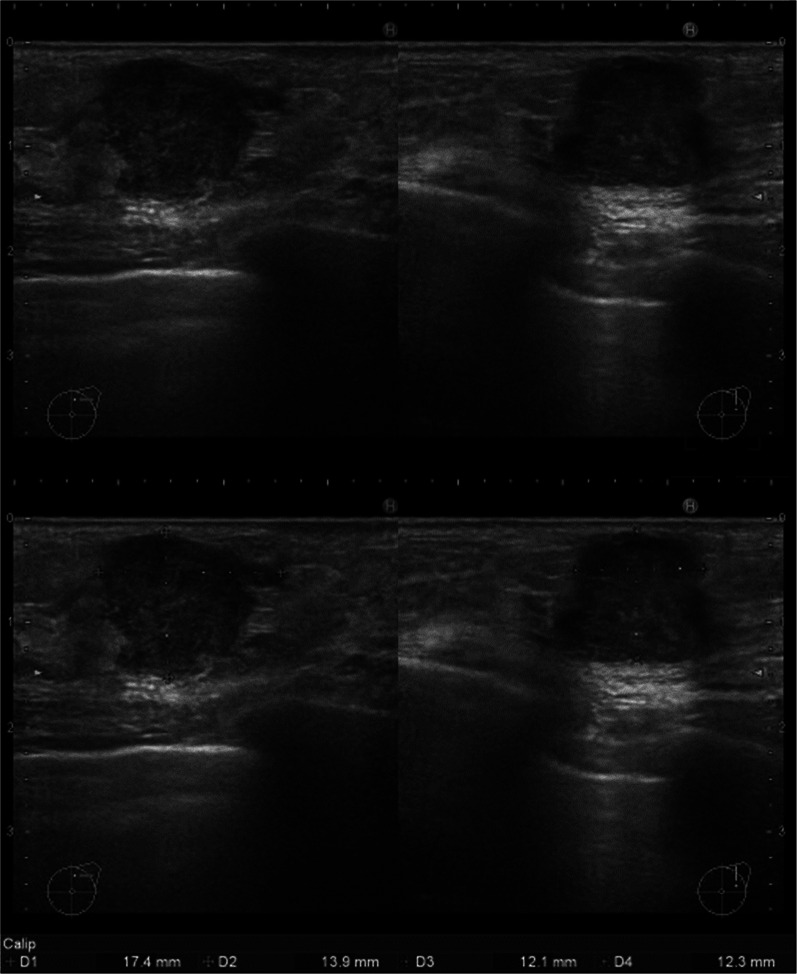
Ultrasound showing a 17.4 × 13.9 × 12.1-mm lobulated mass in the left breast

**Fig. 2:**
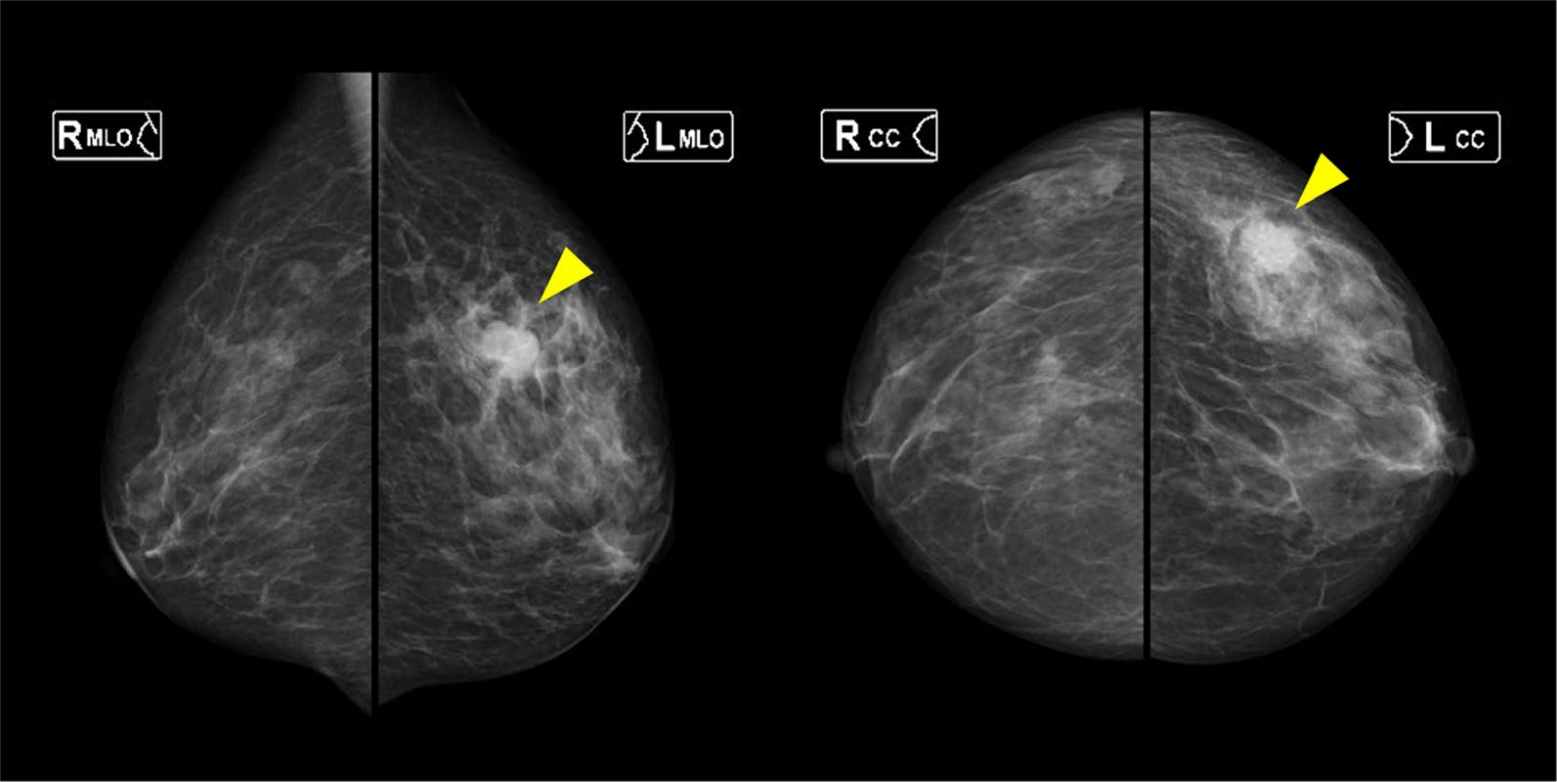
Mammography showing a microlobulated mass on the upper-outer region of the left breast (arrowhead)

**Fig. 3:**
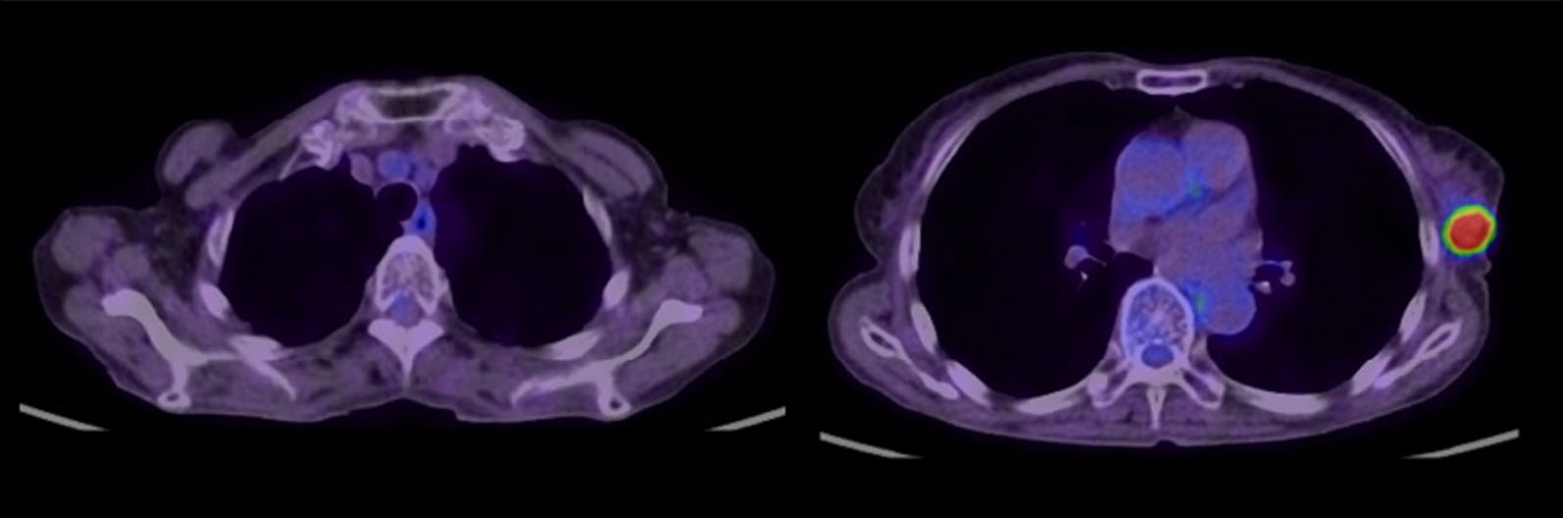
PET–CT scan findings. Left: no lymph node metastasis is suspected. Right: abnormal accumulation is only seen in the left breast

On palpation, there was a 20-mm hard mass at the 1 o'clock position in the left breast. Ultrasonography (US) showed a 17.4-mm lobulated mass at the same location (Fig. 1). Mammography revealed a microlobulated mass on the upper-left side (Fig. 2). The patient could not undergo contrast-enhanced computed tomography (CT) and magnetic resonance imaging because of poor kidney function. Positron emission tomography–CT showed abnormal accumulation only in the left breast, and no lymph node or distant metastasis was suspected (Fig. 3).

**Fig. 4:**
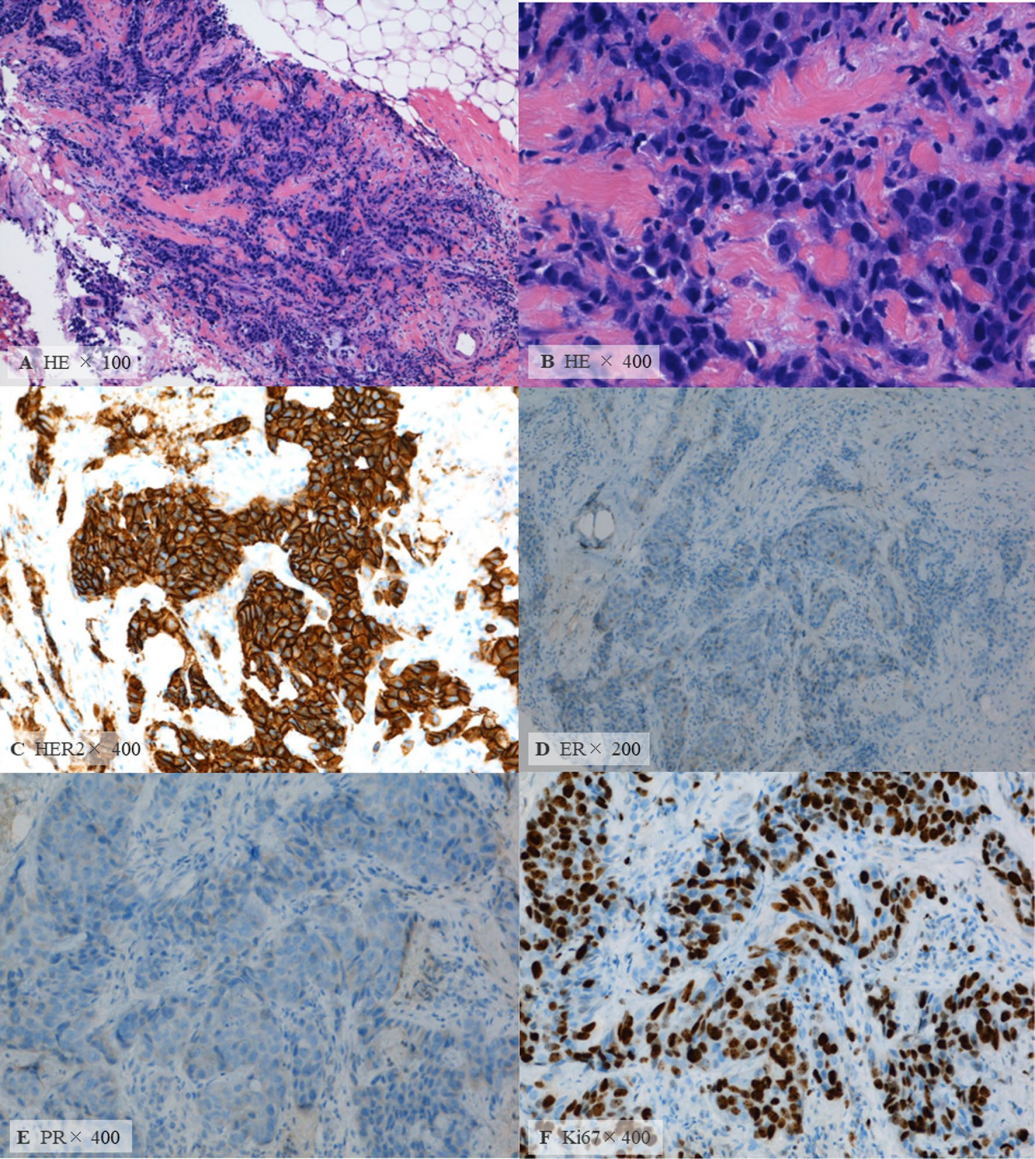
Preoperative histopathological findings. **A**, **B** Atypical cells with a high degree of nuclear atypia proliferated in fullness with stromal fibrosis (hematoxylin and eosin [HE] stain). **C**–**F** Immunohistochemistry study for HER2 (**C**), estrogen receptor (ER) (**D**), progesterone receptor (PR) (**E**), and anti-Ki67 antibody (Ki67) (**F**). The original magnifying powers were as follows: (**A**) × 100, (**B**) × 400, (**C**) × 400, (**D**) × 200, (**E**) × 400, and (**F**) × 400

The pathological diagnosis was invasive ductal carcinoma. The tumor was a high-grade cancer with small nuclei and high nuclear atypia (Grade 3). The subtype was hormone receptor-negative and HER2-positive (Immunohistochemistry: 3 +) (Fig. 4). The examination found that her left breast cancer was HER2-enriched type, T1N0M0, stage I.

The surgery was scheduled a month later, but the patient had concerns regarding the potential for the cancer to progress during the waiting period before the surgery and wanted to receive some kind of drug therapy. We did not recommend neoadjuvant chemotherapy to the patient because of her clinical stage. We also explained that we planned adjuvant trastuzumab monotherapy according to the RESPECT trial, but there was no evidence for its use as neoadjuvant therapy. Despite this, the patient insisted on receiving neoadjuvant trastuzumab. Thus, we decided to use trastuzumab monotherapy as neoadjuvant therapy for two cycles, which could be administered before the surgery date.

**Fig. 5:**
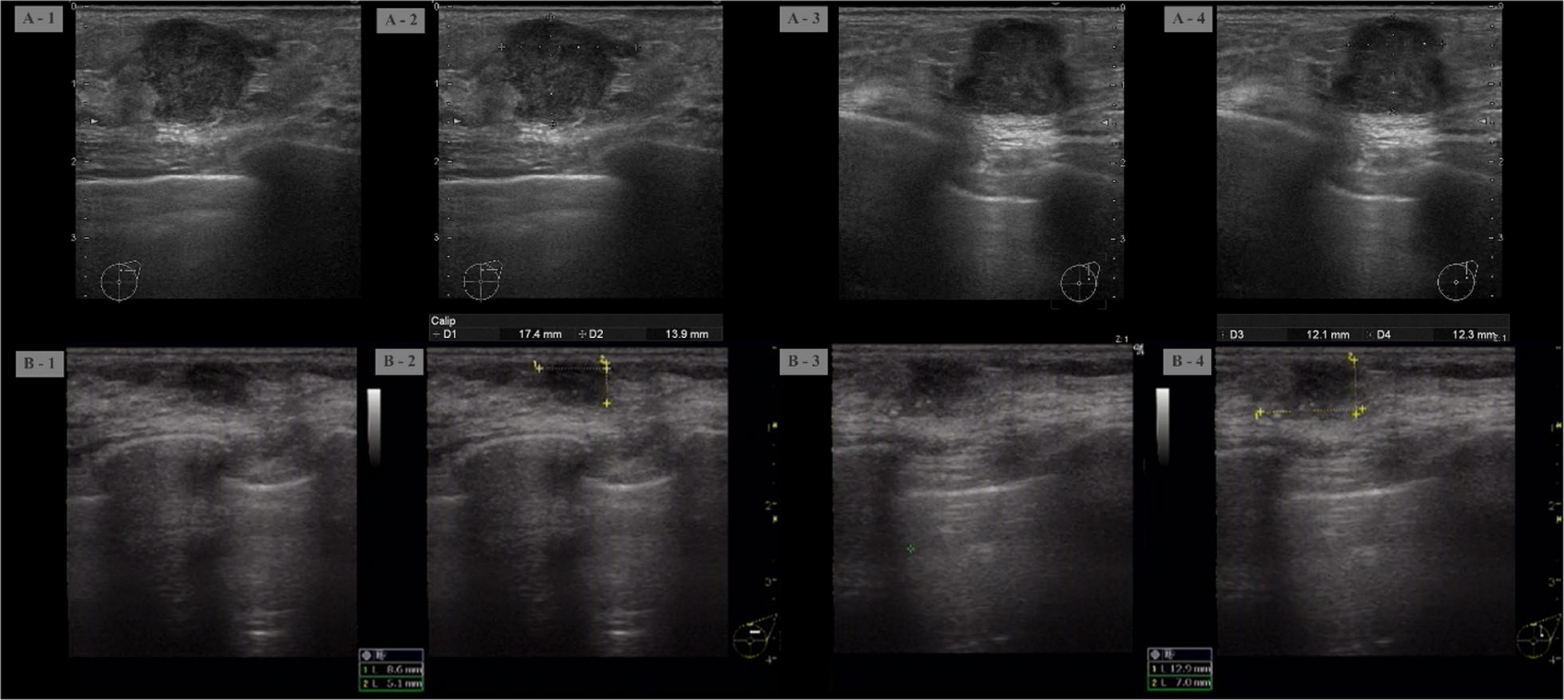
Ultrasound findings pre- and post-trastuzumab therapy. **A** At the time of diagnosis. **B** After a single dose of trastuzumab monotherapy. Tumor diameter appeared to have shrunk overall

Immediately after the first treatment, the patient felt that the tumor was getting smaller. However, severe diarrhea and fatigue occurred as side effects. Therefore, the second cycle of trastuzumab monotherapy could not be administered prior to the surgery. The day before surgery, US revealed that the sum of tumor diameters had shrunk by approximately 40%, which was judged to be a partial response (Fig. 5).

Breast-conserving surgery and sentinel lymph node biopsy were performed, and the patient was discharged without complications.

**Fig. 6:**
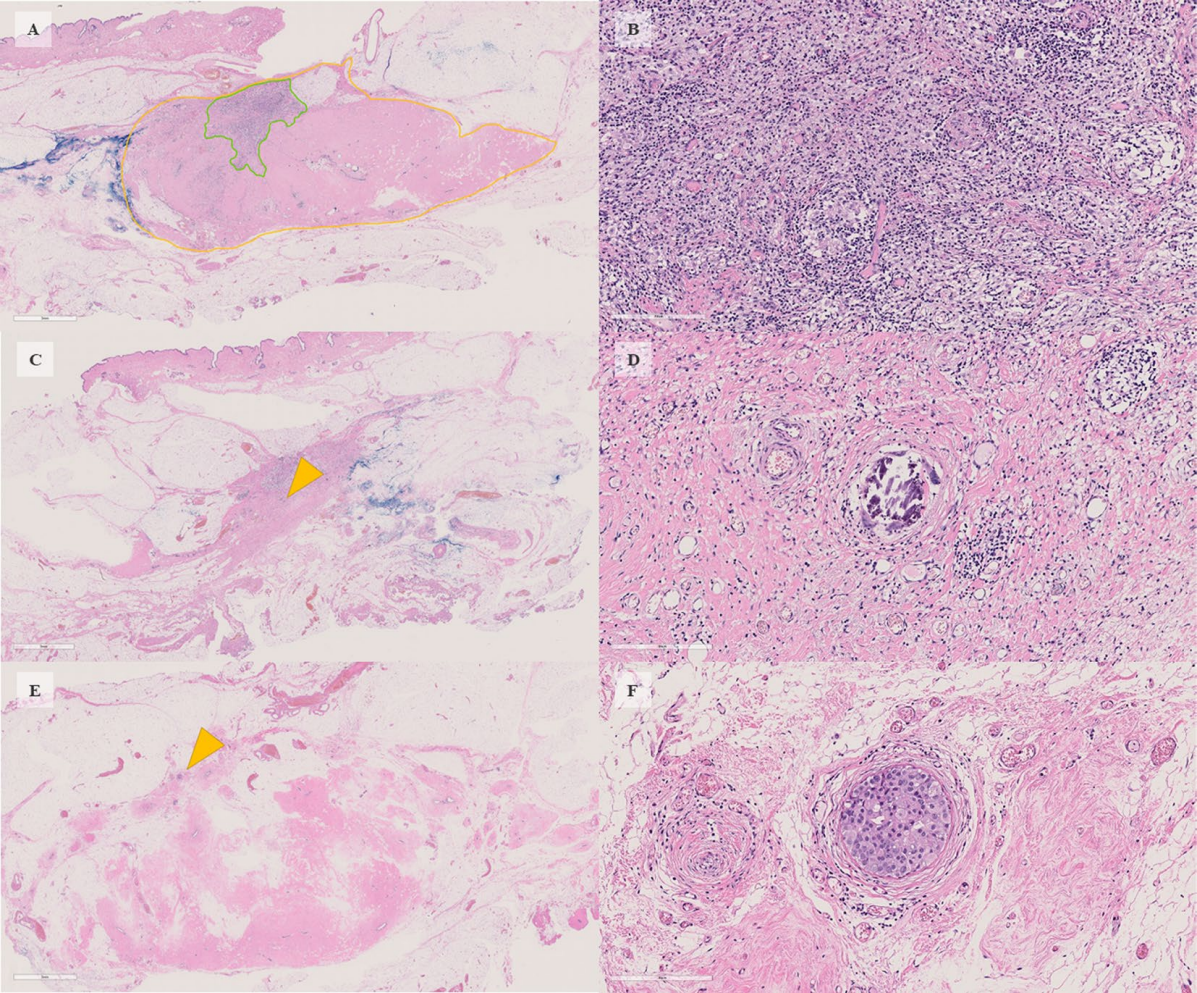
Postoperative histopathological findings. **A**, **B** Main slice of the tumor. The yellow line refers to the fibrosed area where the invasive carcinoma was thought to have been located. The green line refers to the area scarred and infiltrated with inflammatory cells (**A**: HE × 0.8; **B**: HE × 18.6 in the green area). **C**, **D** Outer slice of the tumor. Similar to the US findings, there was calcification within the tumor (yellow arrow) (**C**: HE × 0.8; **B**: HE × 20). **E**, **F** Inner slice distant from the tumor. A ductal carcinoma-in situ component was identified by chance in one glandular duct (yellow arrow), but there was no obvious residual invasive carcinoma, and the surgical margin was negative (**E**: HE × 0.8; **F**: HE × 20)

Surgical pathology specimens showed noninvasive ductal carcinoma in only one mammary duct but no evidence of residual invasive carcinoma. The area where the invasive cancer was located had collagen fiber tears and edematous areas with inflammatory cell infiltration (Fig. 6). These results indicate pCR. The surgical margins were negative for carcinoma, and there was no lymph node metastasis; hence, the lymph node staging was ypN0(sn) (0/1).

Considering the patient’s progress, her severe diarrhea, and her desire to keep her hospital visits to a minimum, we removed adjuvant drug therapy and whole breast irradiation from her treatment plan. Therefore, the patient received no treatment thereafter and was followed up every 6 months with breast palpation, mammography, and tumor marker evaluations (carcinoma antigen 15-3 and carcinoembryonic antigen). No recurrence was observed at 1 year and 6 months postoperatively.

## Discussion

HER2-positive breast cancer accounts for 20–30% of all breast cancers. The prognosis is poor owing to its higher malignant potential compared to that of luminal cancer [2]. However, the advent of molecular-targeted drugs has led to a high therapeutic efficacy. Recently, the RESPECT [8] and WSG-ADAPT trials [9] examined the omission of chemotherapy in elderly patients.

Trastuzumab treatment involves antibody-dependent cellular cytotoxicity (ADCC), which is the primary mechanism of trastuzumab. ADCC is a cell-mediated immune response, in which immune cells induce cell death when specific antibodies are attached to cell membranes. Trastuzumab binds to HER2 receptors on the cell surface, and immune cells, such as natural killer cells, which possess Fcγ receptors and bind to the Fc region of trastuzumab, release various cytotoxic molecules to kill the target cells. In addition, the inflammatory environment induced by ADCC stimulates other immune populations, leading to the emergence of tumor-specific T cells, which may produce a "vaccine effect". This suggests that trastuzumab may be effective as a single agent in patients with breast cancer that overexpresses HER2 [10]. However, there have been no case reports of pCR after a single dose and single-agent application of trastuzumab.

Although very rare, there have been reports of biopsy trauma and other events triggering spontaneous regression (SR) of the tumor. SR is defined as “the partial or complete disappearance of a tumor in the absence of any treatment capable of regression” [11, 12]. While the possibility of SR cannot be completely ruled out in this case, preoperative trastuzumab monotherapy is by no means an ineffective treatment. We also consider that trastuzumab contributed to tumor shrinkage, as the timing of the patient’s realization of tumor shrinkage was immediately after administration.

With the dramatic effects thought to be due to trastuzumab, severe and prolonged diarrhea occurred as a side effect. Diarrhea is a representative side effect of pertuzumab, another HER2 inhibitor used in conjunction with trastuzumab, but is observed less frequently with trastuzumab alone. The mechanism of action of trastuzumab is unclear, and questions remain as to what mechanism causes diarrhea and whether it correlates with tumor shrinkage; however, trastuzumab is effective in some patients.

Currently, genetic polymorphisms of the Fcγ receptor are known to influence the effect of trastuzumab, with some genotypes strongly inducing ADCC and being related to clinical outcomes [13]. Further research is needed to detect targets that would respond well to molecular-targeted therapy without chemotherapy.

If it is possible to identify patients who are more likely to respond to trastuzumab in the future, the range of options for de-escalation therapy without chemotherapy in elderly patients who are concerned about the side effects of chemotherapy will be expanded.

## Conclusion

In this case, we encountered a patient with HER2-positive breast cancer who achieved pCR with a single-agent application and single dose of trastuzumab before surgery. It is possible that some patients with HER2-positive breast cancer will have a significant response to trastuzumab, and one option may be to consider de-escalation therapy without chemotherapy, for example, in elderly patients who wish to avoid side effects as much as possible.

## Data Availability

The data included individual patient data. Additional data are available from the corresponding authors upon reasonable request.

## Abbreviations

HER2: Human epidermal growth factor receptor type 2
pCR: Pathological complete response
US: Ultrasonography
CT: Computed tomography
ADCC: Antibody-dependent cellular cytotoxicity
SR: Spontaneous regression
